# The Relationships between Compulsive Internet Use, Alexithymia, and Dissociation: Gender Differences among Italian Adolescents

**DOI:** 10.3390/ijerph20146431

**Published:** 2023-07-22

**Authors:** Alessandro Germani, Antonella Lopez, Elvira Martini, Sara Cicchella, Angelo Maria De Fortuna, Mirella Dragone, Barbara Pizzini, Gina Troisi, Raffaele De Luca Picione

**Affiliations:** 1 Faculty of Law, Giustino Fortunato University, 82100 Benevento, Italy; a.germani@unifortunato.eu (A.G.); or antonella.lopez@uniba.it (A.L.); e.martini@unifortunato.eu (E.M.); s.cicchella@unifortunato.eu (S.C.); m.dragone@unifortunato.eu (M.D.); b.pizzini@unifortunato.eu (B.P.); g.troisi@unifortunato.eu (G.T.); 2 Department of Educational Sciences, Psychology, Communication, University of Bari, 70122 Bari, Italy; 3 Department of Communication Sciences, Humanities and International Studies (DISCUI), University of Urbino, 61029 Urbino, Italy; angelomariadefortuna@gmail.com

**Keywords:** adolescence, internet, alexithymia, dissociation, age, gender, mediation, moderation

## Abstract

Internet Gaming Disorder, Internet Addiction, Problematic Internet Use and Compulsive Internet Use cause distress and significant impairment in important areas of a person’s functioning, in particular among young people. The literature has indicated that males show higher levels of problematic internet use than females. People can use the internet to avoid or alleviate negative affects; in fact, problematic internet use is associated with alexithymia and dissociation. Few studies have focused on the different stages of adolescence, gender differences, and the relationships between the aforementioned variables. This research aims to fill this gap. Five hundred and ninety-four adolescents aged between 13 and 19 filled in the Compulsive Internet Use Scale, the Toronto Alexithymia Scale, the Adolescents Dissociative Experiences Scale, and other ad hoc measures. Surprisingly, females reported higher compulsive internet use compared with males. Moreover, they referred more difficulties/symptoms and greater levels of alexithymia than males. No differences across the stages of adolescence were found. Different strengths in the relationships between variables were found according to gender. Moderated mediation analyses indicated that dissociation is an important mediator in the relation between alexithymia and Compulsive Internet Use only among females. This study shed new light on gender differences around problematic internet use and some related risk factors, in order to identify and develop prevention and treatment programs to face this topical and relevant issue.

## 1. Introduction

Internet Gaming Disorder, Internet Addiction, Problematic Internet Use, and Compulsive Internet Use are different terms and concepts used to classify and/or describe conditions of internet use which cause distress and significant impairment in important areas of a person’s functioning [1,2,3,4,5,6,7]. These conditions relate in particular to young people (adolescents and young/emerging adults) [8].

Young/emerging adults (aged 18–25 years) have the higher prevalence of any mental illness compared to adults (aged 26–49 years) and those aged 50 and older [9,10]. Adolescents (aged 12–19 years) have the highest prevalence of any mental illness compared to other stages of life. It was estimated that about 50% of adolescents have some form of mental disorder, of which about over 20% have severe impairment and/or distress. Overall, the global onset of the first mental disorder has a peak/median age at onset of 14.5/18 years across all mental disorders. Adolescence can be divided into early adolescence (12–14 years), middle adolescence (15–16 years), and late adolescence (17–19 years). Lifetime prevalence of any mental illness increases during these three stages of adolescence [9,10,11].

Therefore, among young people, adolescents are those who require major attention as to their psychological maladjustment.

In recent years, with the increase in internet use, adolescents are more exposed to the risk of problematic internet use [12]. The literature has indicated that males report a higher level of internet addiction than females [1,2,6], but it is not known if there are differences within the different stages of adolescence. Adolescents can use the internet to avoid or alleviate negative affects, as a tentative way to regulate emotions. In line with this, problematic internet use in young adults and adolescents is associated with alexithymia and dissociation [13,14,15,16]. In other words, it seems that Internet Addiction/Compulsive Internet Use can result from and lend itself to dissociative reactions as strategies to alleviate painful emotions, due to a difficulty to regulate emotions (alexithymia). Both alexithymia [17,18] and dissociation [19,20] decrease during adolescence.

Although some studies have compared Internet Addiction/Compulsive Internet Use and/or alexithymia and/or dissociation levels between different stages of adolescence or between males and females, few studies have tested all these variables and differences in the same study.

Furthermore, even though research has found a positive association between alexithymia and dissociation, and their role on addiction, very few studies have tested the potential role of dissociation as a mediator in the relation between alexithymia and Internet Addiction among emerging adults/adults/elderly [16]. No study has focused on adolescents.

Finally, given the above, no studies have investigated if there are different patterns/relationships between alexithymia, dissociation, and problematic internet use between males and females.

This research aims to fill the aforementioned gaps.

In brief, first of all, it is very important to examine in depth gender differences among adolescents in terms of internet use, alexithymia, and dissociation, and those related to the different stages of adolescence. In other words, are there differences in problematic internet use, alexithymia, and dissociation between males and females, as well as between adolescents of different ages? Second, problematic internet use is associated with alexithymia and dissociation, with the latter being able to partially explain the role of alexithymia as a predictor of internet addiction. However, since there are many gender differences around internet use, alexithymia, and dissociation, it is not known if there are differences in the relationships between these variables according to gender. That is to say, do alexithymia and dissociation have the same role in problematic internet use among males and females?

### 1.1. Problematic Internet Use and Adolescence

#### 1.1.1. Problematic Internet Use

The latest version of the International Statistical Classification of Diseases and Related Health Problems (ICD-11) includes Gaming Disorder, predominantly online (code 6C51.0) [6]. The Gaming Disorder (code 6C51) classified by the ICD-11 “is characterized by a pattern of persistent or recurrent gaming behavior (‘digital gaming’ or ‘video-gaming’), which may be online (i.e., over the internet) or offline, manifested by: 1. impaired control over gaming (e.g., onset, frequency, intensity, duration, termination, context); 2. increasing priority given to gaming to the extent that gaming takes precedence over other life interests and daily activities; and 3. continuation or escalation of gaming despite the occurrence of negative consequences. The pattern of gaming behaviour may be continuous or episodic and recurrent. The pattern of gaming behaviour results in marked distress or significant impairment in personal, family, social, educational, occupational, or other important areas of functioning. The gaming behaviour and other features are normally evident over a period of at least 12 months in order for a diagnosis to be assigned, although the required duration may be shortened if all diagnostic requirements are met and symptoms are severe” [6]. Gaming disorder predominantly online is different from Gambling disorder predominantly online (6C50.1), Hazardous gambling (QE21), and Hazardous gaming (code QE22), and it cannot be explained by bipolar disorders.

The Diagnostical and Statistical Manual of Mental Disorders (DSM-5 and DSM-5-TR), besides Gambling Disorder (681), includes Internet Gaming Disorder among the conditions which need further studies. More specifically, Internet Gaming Disorder requires the following diagnostic criterion. A significant impairment or distress in several aspects of a person’s life with the presence of five (or more) of the following symptoms: Preoccupation with gaming on the internet; withdrawal symptoms when gaming is taken away or not possible (sadness, anxiety, irritability); tolerance, the need to spend more time gaming to satisfy the urge; inability to reduce playing, unsuccessful attempts to quit gaming; giving up other activities, loss of interest in previously enjoyed activities due to gaming; continuing to game despite psychosocial problems; deceiving family members or others about the amount of time spent on gaming; the use of gaming to avoid or relieve negative moods, such as hopelessness, guilt, anxiety; risk, having jeopardized or lost a job or relationship due to gaming [1,2].

The Second Version of the Psychodynamic Diagnostic Manual (PDM-2), in Section II dedicated to Adolescents, describes Internet Addiction Disorder (code SA94). In this Diagnostic Manual, Internet Addiction Disorder is also called Internet Addiction or Compulsive Internet Use. The PDM-2 includes different behaviors of excessive internet use, such as gaming online, social networking, e-mails, online pornography, and shopping online, which compromise personal functioning and are associated with loss of control, tolerance, and withdrawal [3].

Therefore, while ICD-11 and DSM-5-TR focus on problematic internet use related to gaming online, the PDM-2 considers different behaviors of problematic internet use. All the aforementioned diagnostic systems report that these disorders commonly co-occur with other mental disorders, such as Disorders Due to Substance Use, Mood Disorders, Anxiety or Fear-Related Disorders, Attention Deficit Hyperactivity Disorder, Obsessive Compulsive Disorder, and Sleep–Wake Disorders [1,2,3,6]. A recent meta-analysis suggested the existence of some risk factors for problematic internet use related to gaming online, such as male gender, loneliness, depression, anxiety, stress, impulsivity, hostility/aggressivity as a trait, gaming time, escape motivation, or excessive use of social networks [21].

Both Gaming Disorder predominantly online (ICD-11) and Internet Gaming Disorder (DSM-5-TR), as well as Internet Addiction Disorder (PDM-2), are classifications subsequent to the description of Internet Addiction made by Young [7], who tried to identify the differences between normal and addicted internet usage (i.e., technological overuse, computer dependency, obsessive video game playing), emphasizing the similarities of the latter condition with Pathological Gambling and substance abuse. In this view, Internet Addiction was defined as an impulse control disorder that does not involve an intoxicant, characterized by thoughts, emotions, and behaviors related to internet use, such as preoccupation; need to use to escape from problems or relieve a dysphoric mood; unsuccessful efforts to control, cut back, or stop with feelings of restlessness, moodiness, depression, and irritation; and problems with relationships, jobs, and education.

Some authors prefer the term Compulsive Internet Use to Internet Addiction, because they believe that one is not addicted to the internet itself, but rather to certain online activities, resulting in compulsive internet use [4]. In defining Compulsive Internet Use, loss of control, preoccupation, feelings of depression, loneliness, and tension, as well as intrapersonal and interpersonal conflict and withdrawal symptoms seem to be crucial. Tolerance and lying about involvement seem to play a secondary role [4,22].

Other authors have argued that the term Problematic Internet Use is better than Internet Addiction and Compulsive Internet Use, which emphasize addiction or impulse control. Problematic Internet Use, instead, is descriptive and non-interpretive [5,15]. Although there is not agreement on terms, definitions, conceptualizations, etc. [23], to date, as Peter Mitchell wrote some years ago, “Even though there is still disagreement about whether internet addiction is a real diagnosis, there is no doubt that the compulsive use of the internet, like other dependencies, has material as well as psychological dangers” [24].

Available data suggest that adults have lower prevalence rates than adolescents [1,2,8]. Among adolescents, Gaming Disorder has been associated with high levels of externalizing and internalizing problems. Adolescents with Gaming Disorder may be at increased risk for academic underachievement, school failure/drop-out, and psychosocial and sleep problems [1,2,6].

#### 1.1.2. Problematic Internet Use: Age and Gender Differences in Adolescence

Adolescence is characterized by very important changes at the bodily, sexual, emotional, cognitive, interpersonal, and social levels. The development of identity, personality, autonomy, and independence, as well as the capacity to relate, are the core aspects of this stage of life [25].

Overall, to date, the literature suggests that Internet Gaming Disorder appears to be most prevalent among adolescents and young adult males aged 12 to 20 years. In general, males appear to be more frequently affected by Gaming Disorder and Problematic Internet Use [1,2,6,26,27]. Though less frequently diagnosed with Gaming Disorder than adolescent boys, girls who meet the diagnostic requirements may be at greater risk of developing emotional or behavioral problems. Therefore, besides gender differences in Internet Gaming Disorder prevalence, females require particular attention. With regard to age, no studies have investigated the potential role of stages of adolescence on Compulsive Internet Use. Although adolescents and young adults suffer from Internet Addiction/Compulsive Internet Use more than adults, no studies have yet investigated differences across different stages of adolescence in depth.

With regard to gender, more specifically, Morahan-Martin and Schumacher [28] found in a sample of college students that males were more likely than females to be pathological users, with a ratio of 4:1. Pathological use was determined by responses to 13 questions which assessed evidence that internet use was causing academic, work, or interpersonal problems, personal distress, withdrawal symptoms, or mood-altering use. Scherer [29] tested gender differences among college students and reported that dependent internet users included a significantly larger proportion of men to women than the non-dependent users. Chou and Hsiao [30], among a sample of more that 900 Taiwan college students, found that only three respondents were female students out of a total of 54 internet addiction cases. With regard to adolescence, Di Nicola et al. [31] found no gender differences in the Internet Addiction Test among Italian adolescents and young adults. Ko et al. [32] found gender differences in the Chinese Internet Addiction Scale among Taiwanese adolescents, with males reporting higher levels than females.

Therefore, it seems important to investigate gender differences in more depth, as well as age group differences, among adolescents, with regard to problematic internet use (and not only internet gaming), and take into account that females and males can use internet in different ways (different activities such as gaming, chat, posting and viewing photos, videos, etc.).

### 1.2. Internet Use, Alexithymia, and Dissociation

In the previous sections it was highlighted that people can use the internet to avoid or alleviate negative affects, and they can use it in a compulsive way, showing preoccupation, loss of control, withdrawal, and tolerance related to the internet use [1,2,3,4,5,6,7,15]. In other words, addiction behaviors like Compulsive Internet Use may be considered reactions to dysregulated emotions, or rather a coping strategy to compensate for emotional regulation deficits, namely, to regulate emotions. Recent reviews [12,33] found strong associations between problematic internet use and emotion dysregulation, considered and measured above all by the Difficulties in Emotion Regulation Scale [34] and the Emotion Regulation Questionnaire [35]. The literature suggests that high levels of alexithymia impair the appropriate selection of emotion regulation strategies [36]. In this context, Yang et al. [33] mentioned that the construct of emotional dysregulation has many facets and dimensions, such as alexithymia, that should be better investigated in relation to addiction behaviors.

The concept of alexithymia has received increasing levels of attention. This concept was formulated by Sifneos in 1973 [37]. It describes people with emotions (biological components of affects) but with a deficit in ability to represent emotions, namely through psychological components of affects, such as images, thoughts, fantasies, dreams. In other words, a difficulty in experiencing feelings. Alexithymia is considered a difficulty in elaborating visceral information, sensations, and perceptions; processing and regulating emotions; representing, symbolizing, and thinking (consciously and unconsciously); and naming emotions. In other words, alexithymia can be considered a difficulty in integrating emotions and cognition, and experiencing feelings without denying/avoiding/discharging/acting out emotions. More specifically, alexithymia is defined as an incapacity to discriminate between physical sensations and feelings, to distinguish different affective states, and to verbalize them. Alexithymia is described as being composed by the following dimensions: difficulty in identifying feelings; difficulty in communicating feelings to others; and externally oriented thinking (operational thinking) [38]. Alexithymia is not a diagnostic category, it is not a disorder, and it is not only related to psychosomatic conditions, but it can be viewed as an aspect of personal functioning that has a role in many clinical conditions, such as medical and psychological disorders [39,40]. Studies have found that alexithymia has a role in Internet Addiction/Problematic Internet Use [15,41].

Besides alexithymia, dissociation has a role in internet addiction as a way to escape from negative emotional states [13,14,16,26,27,31]. Dissociation is considered to be a lack of integration or disaggregation due to extreme emotional arousal, as well as a defense mechanism, namely, a mental process which can vary along a continuum from a normal to pathological level, from adjustment to maladjustment [42]. Putnam [42] identified the defense role of dissociation in four main aspects: automation; alteration and alienation of Self; compartmentalization; and protection from suffering that cannot be processed. From a phenomenological point of view, dissociation refers to three different meanings [3]: dissociation of some mental functions (a disconnection between a person’s thoughts, memories, feelings, actions or sense of who he or she is); dissociation as depersonalization/derealization (experiences of unreality or detachment from one’s mind, self or body/one’s surroundings); dissociation as multiplicity (e.g., plurality of consciousness, dissociative identity disorder). Referring to the nosology, the ICD-11 [6] distinguishes between Dissociative Neurological Symptom Disorder, Dissociative Amnesia, Trance Disorder, Possession Trance Disorder, Dissociative Identity Disorder, Partial Dissociative Identity Disorder, and Depersonalization–Derealization Disorder. The DSM-5 [1,2] instead classifies Dissociative Identity Disorder, Dissociative Amnesia, and Depersonalization/Derealization Disorder. The PDM-2 [3], considering the specificity of dissociation during adolescence, reports that adolescents can have a variety of dissociative symptoms as a lack of continuity in mental functioning, which can be associated with many different evolutive or clinical conditions. Research has found a positive association between dissociation and Internet Addiction [13,14,16,31].

Dissociative reactions are manifestations of defense mechanisms used to alleviate painful emotions, while alexithymia is an emotional dysregulation. Dissociation and alexithymia can be considered distinctive but overlapping phenomena. Research has identified it both in adulthood and in adolescence. The strongest association is between the difficulty of identifying feelings (one dimension of alexithymia) and dissociation. Scholars have argued that alexithymic characteristics may contribute to dissociation [43,44,45,46,47].

The literature [15] suggests that alexithymia may play a role in pathogenesis and maintenance of Internet Addiction, with a facilitating role for dissociation. Craparo [13] found significant correlations between Internet Addiction and both alexithymia and dissociation among Italian students aged between 18 and 21 years. A recent study [16] found that dissociation partially explained the relationships between alexithymia and problematic online gambling, among a sample of emerging adults, adults, and the elderly.

No studies have tested the role of dissociation in the relationships between alexithymia and problematic internet use among adolescents.

Referring to gender and age differences in levels of alexithymia and dissociation, some studies [17,18] have suggested that alexithymia decreases during adolescence, and other studies have suggested that normative dissociation reaches its peak during latency years, declining during adolescence [19,20]. Referring to gender, research has suggested that adolescent females report a higher difficulty in identifying and expressing feelings than males, while there were no significant differences in the levels of dissociation [46]. Despite everything that has been said, so far as is known, no studies have investigated if alexithymia and dissociation have the same role in problematic internet use among male and female adolescents.

### 1.3. The Present Study

This study aimed to evaluate among adolescents, distinguished by gender and age, (a) problematic internet use, alexithymia, and dissociation levels; (b) the correlations between internet use, alexithymia, and dissociation; and (c) the role of dissociation in the relationship between alexithymia and problematic internet use.

Based on the literature [1,2,6,26,27,46], it was hypothesized that males would report more problematic internet use than females, while females would report more alexithymia than males. No gender differences were expected in dissociation levels. Moreover, it was expected that both alexithymia and dissociation would decrease with age.

Regarding the relationships between the main variables, it was hypothesized that alexithymia and dissociation were positively associated with problematic internet use, with dissociation playing a mediation role in the relationship between alexithymia and problematic internet use [13,14,15,16,31,43,44,45,46,47]. No hypothesis was advanced for gender as a moderator.

## 2. Materials and Methods

### 2.1. Participants and Procedures

The research was implemented in a medium-sized town in southern Italy during the month of May 2022. Participants were recruited from high schools. Parental signed consent was required and collected for students under 18, while students aged 18 and over signed the consent by themselves. The study was conducted in accordance with the 1964 Helsinki Declaration and its later amendments or comparable ethical standards, the ethical code of the Italian Psychology Association (AIP) (http://www.aipass.org/node/26, accessed on 10 January 2021), and the Italian Code concerning the protection of personal data (Legislative decree No. 196/2003). Participants signed informed consent. No incentive was given. The research project was previously submitted and then approved by the Research Committee of Giustino Fortunato University (Benevento, Italy) through the protocol of the Academic Senate on 2 February 2021.

Data were collected through convenience sampling, adopting the following inclusion criteria: (a) signed the informed consent, (b) completed the entire survey form, (c) aged between 12 and 19 (adolescents). Data were collected through the use of an online survey (Google Forms) that the participants filled in during school time.

A total of 624 Italian high school students participated in the study. Participants who did not meet one or more inclusion criteria were removed. The final sample comprised 594 adolescents aged between 13 and 19 (Mean age = 16.04; Sd = 1.45). Participants were 47.6% female and 52.4% male, of whom 20.4% were early, 40.0% were middle, and 39.6% were late adolescents.

### 2.2. Measures

#### 2.2.1. Internet Use. A Set of Items That Assess

Time spent on the Internet: Participants were required to indicate on a 4-point scale (from 0 = “up to half an hour” to 3 = “more than two hours”) the average amount of time spent on the internet every day.

Activities on the Internet: Participants were asked to indicate on a 4-point scale (from 1 = “not at all” to 4 = “extremely”) how connected they are to the internet when doing the following activities: Listening to music; watching videos/series; looking at images, photos, stories; gaming; searching for information; studying; chatting with friends/acquaintances; sending/receiving email; navigating without a destination; shopping; making new friends or dating.

#### 2.2.2. Symptoms/Difficulties

A set of 22 items (“Have you noted recently the following manifestations, behaviors or feelings?”) were graded on a 4-point Likert scale from 1 (“never”) to 4 (“always”) to assess a series of symptoms/difficulties (e.g., sleeping, eating, physical, anxiety, attention, depressive, addiction). The total score was the mean of all items and ranges between 1 and 4. Higher scores indicate greater difficulty. Cronbach’s alpha was 0.94.

#### 2.2.3. Satisfaction with Life

A set of 8 items (“How much are you satisfy in each of the following aspects of your life?”) were ranked on a 4-point Likert scale from 1 (“not at all”) to 4 (“extremely”) to assess satisfaction with some aspects of life (general, affects, society, friendships, family, school, leisure, future). The total score was the mean of all items and ranges between 1 and 4. Higher scores indicate a greater satisfaction with life. Cronbach’s alpha was 0.85.

#### 2.2.4. Compulsive Internet Use

Compulsive Internet Use Scale (CIUS-14) [4,48,49]. CIUS-14 is a self-reported measure with items rated on a 5-point Likert scale (from 0 = “never” to 4 = “very often”). The 14 items represent the core elements of compulsive or addictive internet use, and they comprise one factor. The total score ranges between 0 and 56. The higher the score is, the higher the Compulsive Internet Use will be. The cut-off is >21. Cronbach’s alpha was 0.94.

#### 2.2.5. Alexithymia

Toronto Alexithymia Scale (TAS-20) [38,50]. TAS-20 is a self-reported measure with items rated on a 5-point Likert scale (from 1 = “strongly disagree” to 5 = “strongly agree”). The 20 items comprise 3 dimensions: difficulty identifying feelings; difficulty describing feelings to others; and externally oriented style of thinking. The total score ranges between 20 and 100. The higher the score is, the higher the alexithymia will be. The TAS-20 cut-off points are: ≤51 for non-alexithymia; 52–60 for possible alexithymia; ≥61 for alexithymia. Cronbach’s alphas were 0.80 for total score, 0.90 for difficulty identifying feelings, 0.74 for difficulty describing feelings to others, and 0.32 for externally oriented style of thinking.

#### 2.2.6. Dissociation

Adolescence-Dissociative Experience Scale (A-DES) [51,52,53]. A-DES is a self-reported measure in a version for adolescents, with items rated on a 11-point Likert scale (from 0 = “never” to 10 = “always”). The 30 items comprise 4 dimensions: dissociative amnesia; absorption and imaginative involvement; depersonalization and derealization; and passive influence. The total score is equal to the mean of all the items and ranges between 0 and 10. The higher the score is, the higher the dissociation will be. The A-DES cut-off is <4. Cronbach’s alpha was 0.95 for total score, 0.84 for dissociative amnesia, 0.77 for absorption and imaginative involvement, 0.90 for depersonalization and derealization, and 0.79 for passive influence.

### 2.3. Data Analysis

Normality for all the measures was tested, including skewness and kurtosis, with values between −2.0 and 2.0 [54].

In order to describe the sample, preliminary analyses were carried out. Descriptive statistics were carried out for gender and age group. A Chi-square test was run to verify the distribution of gender and age group. Then, the effects of gender, age group, and their interaction were tested on Internet Use, Symptoms/Difficulties, and Satisfaction with Life. All the scores related to the items of Internet Use were compared using Multivariate Analysis of Variance (MANOVA) with gender and age groups as between-subject variables, testing their interaction. Finally, two separate ANOVAs were run to compare Symptoms/Difficulties scores and Satisfaction with Life scores, with gender and age groups as between-subject variables, testing their interaction. Effect size was measured using partial eta-squared, with 0.0099, 0.0588, and 0.1379 being small, medium, and large effects, respectively [55]. Therefore, only significant differences with at least small effect sizes (*ŋp*^2^ > 0.0099) were taken into account, because differences showing effect sizes lower than 0.0099 could be trivial.

Then, gender and age group differences were tested on Problematic Internet Use, alexithymia, and dissociation. All the scores were compared using MANOVA with gender and age group as between-subject variables, testing their interaction. A series of chi-square tests was run to verify the distribution of the categories of alexithymia, dissociation, and Compulsive Internet Use between gender.

Correlations between Problematic Internet Use, alexithymia, and dissociation were carried out separately for females and males. Effect size was interpreted according to Cohen [56], with correlation coefficients of 0.10, 0.30, and 0.50 representing small, medium, and large effect size, respectively. Thus, significant correlations with Pearson’ s *r* lower than |0.10| were not interpreted because they are considered negligible.

**Figure 1:**
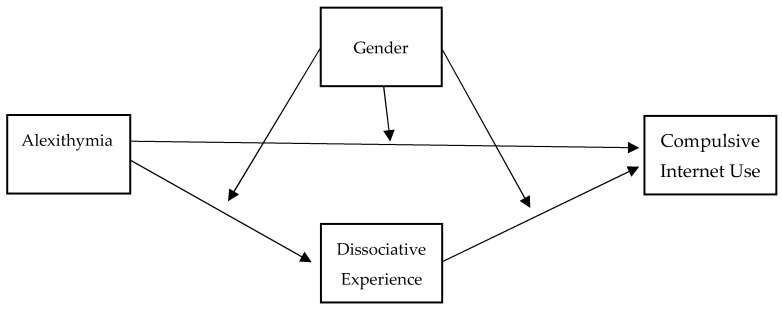
Conceptual diagram of the moderated mediational model.

Two separate simple linear regressions were run to calculate the total effect of TAS-20 on CIUS-14, separately in females and males. To examine the moderated mediational model, according to Germani and colleagues [57], SPSS macro was used (Process, Model 59) [58]. Please, see the conceptual diagram in Figure 1. A variable has a moderation effect when the indirect effect (mediation) of an independent variable on a dependent variable changes at different levels of the moderator [56]. The mediation of A-DES in the relation between TAS-20 and CIUS-14, and the moderation of gender (i.e., Females and Males), were tested by using a 5000 bootstrap sample and 95% confidence intervals (CI). If the confidence interval excluded 0, the effect was significant [58,59]. Gender was inserted in the model as a moderator, testing all possible interactions (TAS-20 × Gender on A-DES, A-DES × Gender on CIUS-14, TAS-20 × Gender on CIUS-14). The proportion mediated (PM = indirect effect/total effect) was calculated.

## 3. Results

### 3.1. Preliminary Results

In Table 1 are reported demographic variables, Internet Use variables, Symptoms/Difficulties, and Satisfaction with Life of the sample, by gender and age group.

The Chi-square test highlighted that the distribution of gender across age groups was equal.

There were no gender and age group differences in the amount of time spent on the internet. With regard to the activities on the internet, the main results of interest for problematic internet use indicated that females spent more time than males viewing images, videos or stories, chatting with friends, and navigating without a destination; while males reported a higher amount of time spent gaming online than females. Moreover, the time spent viewing images, photos or stories, searching for information, sending/receiving emails, shopping, and chatting with friends/acquaintances increased across age groups.

In order to analyze the role of Internet Use on Compulsive Internet Use, two multiple regression analyses for female and male participants were conducted using CIUS-14 score as a dependent variable and Activities on Internet scores as independent variables. Results indicated that viewing images, photos, stories (*b* = 0.145, *t* = 2.12, *p* = 0.035) for females, and watching videos/series (*b* = 0.132, *t* = 2.12, *p* = 0.035), gaming (*b* = 0.122, *t* = 2.06, *p* = 0.040), and navigating without a destination (*b* = 0.148, *t* = 2.52, *p* = 0.012) predicted CIUS-14 score.

Referring to psychological adjustment/maladjustment, Satisfaction with Life decreased and Symptoms/Difficulties increased significantly across the stages of adolescence. Females reported more Symptoms/Difficulties than males.

### 3.2. Gender and Group Age Differences on TAS-20, A-DES, and CIUS-14 Total Scores

The results of the MANOVA revealed a significant multivariate main effect for gender (*Wilks’ λ*= 0.908, *F* (3, 586) = 19.71, *p* < 0.001, *ŋp*^2^ = 0.092), but not for age group (*Wilks’ λ* = 0.981, *F* (6, 1172) = 1.90, *p* = 0.076, *ŋp*^2^ = 0.010) or their interaction (*Wilks’ λ* = 0.991, *F* (6, 1172) = 0.87, *p* = 0.513, *ŋp*^2^ = 0.004). Mean, standard deviation, and the univariate main effects for gender and age groups are shown in Table 2 and Table 3. There were significant and meaningful differences in TAS-20 total score with a medium effect size, and CIUS-14 score with a small effect size, with females reporting higher levels than males. No differences were found for A-DES TS. In line with this, the following percentages were recorded according to the cut-offs of the three measures: Referring to TAS-20, among females: non-alexithymia—28.3%; possible alexithymia—28.6%; alexithymia—43.1%; among males: non-alexithymia—49.8%; possible alexithymia—29.6%; alexithymia—20.6%; (*χ*^2^ (2) = 41.49, *p* < 0.001). Referring to A-DES, among females: normal dissociation—72.8%; pathological dissociation—27.2%; among males: normal dissociation—74.3%; pathological dissociation—25.7%; (*χ*^2^ (1) = 0.17, *p* = 0.682). Referring to CIUS-14, among females: non-compulsive internet use—47.3%; Compulsive Internet Use—52.7%; among males: non-compulsive internet—68.5%; Compulsive Internet Use—31.5% (*χ*^2^ (1) = 27.26, *p* < 0.001).

Since there was not a significant multivariate main effect of age group, or a significant interaction between age group and gender, the age group variable was not considered in the following analyses.

### 3.3. Gender Differences on TAS-20 and A-DES Subscales

The results of the MANOVA revealed a significant multivariate main effect for gender (*Wilks’ λ*= 0.869, *F* (7, 586) = 12.66, *p* < 0.001, *ŋp*^2^ = 0.131). The mean, standard deviation, and univariate main effects for gender are shown in Table 4. There were significant and meaningful difference in all the TAS-20 subscales, with females reporting higher scores in DIF and DDF than males, who in turn reported higher EOT levels. No differences were present in the A-DES subscales.

### 3.4. Correlations between TAS-20, A-DES, and CIUS-14 Dimensions

As shown in Table 5, the TAS-20 TS was positively and significantly associated with the CIUS-14 score with medium effect size, in both groups. Referring to TAS subscales, among females: DIF had a medium effect size; DDF and EOT had a small effect size; among males: DIF and DDF had a medium effect size; EOT was not significantly associated.

TAS-20 TS and A-DES TS were positively and significantly correlated, with a medium effect size, both in females and males. More specifically, DIF was correlated to all the A-DES subscales with a medium effect size in both groups, while DDF and EOT had a small effect size in both groups.

The A-DES TS and subscales were positively and significantly associated with CIUS-14 score, with a medium effect size among females and a small effect size among males.

### 3.5. Moderated Mediation Model with TAS-20, A-DES, and CIUS-14

Before testing the moderated mediational model, the total effect of TAS-20 on CIUS-14 was tested separately in females and males. With regard to females, the model was significant (*F* _(1281)_ = 44.95, *p* < 0.001) and explained about 14% of the variance in CIUS-14 (*R*^2^ = 0.138). TAS-20 had a significant total effect on CIUS-14 (*b* = 0.371, *t* = 6.70, *p* < 0.001). With regard to males, the model was significant (*F* _(1309)_ = 74.08, *p* <0.001) and explained about 19% of the variance in CIUS-14 (*R*^2^ = 0.191). TAS-20 had a significant total effect on CIUS-14 (*b* = 0.440, *t* = 8.60, *p* < 0.001).

The model with TAS-20, gender, and their interaction as predictors and CIUS-14 as the outcome variable was significant (*F* _(3590)_ = 52.19, *p* < 0.001) and explained about 21% of the variance in CIUS-14 (*R*^2^ = 0.209). Overall, TAS-20 had a significant total effect on CIUS-14 (*b* = 0.393, *t* = 10.76, *p* < 0.001) and was not moderated by gender, because the interaction between TAS-20 and gender was not significant (*b* = 0.072, *t* = 0.99, *p* = 0.321).

Next, the effect of TAS-20 on mediator was calculated (see Table 6). The model with TAS-20, gender, and their interaction as predictors and A-DES (mediator) as an outcome variable was significant (*F* _(3590)_ = 46.49, *p* < 0.001) and explained about 19% of the variance in A-DES (*R*^2^ = 0.191). TAS-20 significantly predicted A-DES (*b* = 2.232, *t* = 11.69, *p* < 0.001), but its interaction with gender was not significant (*b* = 0.597, *t* = 1.57, *p* = 0.117).

Finally, the full moderated mediational model was tested (see Figure 1 and Table 6). It accounted for about 23% of the explained variability in CIUS-14 score (*R*^2^ = 0.235) and was significant (*F* _(5588)_ = 36.12, *p* < 0.001). A-DES significantly predicted CIUS-14 scores (*b* = 0.029, *t* = 3.83, *p* < 0.001). TAS-20 showed a direct effect on CIUS-14 (*b* = 0.332, *t* = 8.30, *p* < 0.001). The interaction between TAS-20 and gender was not significant (*b* = 0.132, *t* = 1.66, *p* = 0.096), while the interaction between A-DES and gender was significant (*b* = −0.035, *t* = −2.28, *p* = 0.023). More specifically, TAS-20 had a direct effect on CIUS-14, both in females (*b* = 0.262, *t* = 4.89, *p* < 0.001) and males (*b* = 0.395, *t* = 6.72, *p* < 0.001). Furthermore, TAS-20 had an indirect effect on CIUS-14 as mediated through A-DES in females (*b* = 0.093, *CI* = 0.039, 0.158, *PM* = 25.07%), but not in males (*b* = 0.032, *CI* = −0.024, 0.090, *PM* = 7.27%).

## 4. Discussion

This study was aimed to evaluate differences between gender (females vs. males) and age group (early, middle, and late adolescents) in adolescents with regard to problematic internet use (Compulsive Internet Use), alexithymia, and dissociation levels, as well as the relations between these variables, with particular attention to dissociation as a potential mediator in the effect of alexithymia on problematic internet use. Overall, results indicated that there were important gender differences, while age group was not so important in explaining Compulsive Internet Use, alexithymia, and dissociation levels, as well as the relations between these variables.

The main results were that females reported higher Compulsive Internet Use and alexithymia levels than males. The average dissociation level was similar in these two groups. The results related to Compulsive Internet Use did not confirm the hypothesis based on the literature [1,2,6,26,27], according to which males were supposed to show higher problematic internet use than females. Referring to age group, no significant differences were found. The association between alexithymia and Compulsive Internet Use, as well as the correlation between alexithymia and dissociation, were similar for males and females, showing a positive and significant association with medium effect size. Specifically, the difficulty to identify feelings was the dimension of alexithymia strongly associated with dissociation and Compulsive Internet Use. Besides these similarities, the relation between dissociation and Compulsive Internet Use was stronger among females than males. In fact, dissociation partially explained the effect of alexithymia on Compulsive Internet Use only in the female group. In other words, gender moderated the relation between alexithymia and problematic internet use via dissociation, which was a mediator of this relationship only for females. That is to say, alexithymia played a main role in problematic internet use among adolescents irrespective of gender. Although dissociation levels were similar in females and males, it played a key role in Compulsive Internet Use among females.

### 4.1. Problematic Internet Use, Alexithymia, and Dissociation Levels

In order to analyze the main results more deeply, it is important to note that there were gender differences in the use of the internet. Although there were no gender or age differences in the average amount of time spent every day on the internet, females reported that they used the internet to view images, photos, and stories, chat with friends/acquaintances, and shop online much more frequently than males. The same difference was present across the three stages of adolescence, with increasing age. Moreover, females reported that they navigated the internet without a destination more than males, who in turn played games online much more than females. These results are in line with research that indicated different use of the internet in males and females, as well as with recent studies that indicated a changing trend towards female predominance in excessive internet use [60,61], and are essential to explaining gender differences on Compulsive Internet Use. As said, females reported higher Compulsive Internet Use than males, which was opposite most results present in the literature [28,29,30,32,60]. By using the CIUS-14 cut-off, about a half of female participants can be considered addicted to the internet, compared to one-third of males. Taking into account the activities that participants engaged in on the internet, watching videos/series, viewing images, photos, and stories, gaming online, navigating on the internet without a destination, and making new friends were associated with Compulsive Internet Use. Multiple regression analyses indicated that, for males, navigating on the internet without a destination, watching video/series, and gaming online; and for females, viewing images, photos, and stories, were the activities particularly related to internet addiction. Furthermore, additional evidence has come to our attention regarding the female group: the higher presence of difficulties/symptoms compared to males. Finally, across stages of adolescence, symptoms increased, and Satisfaction with Life decreased.

Regarding the gender and age group differences referring to alexithymia and dissociation, in line with the literature [46], females reported higher alexithymia than males. By looking at the TAS-20 cut-off, over 40% of females exceeded the cut-off for alexithymia, compared to about 20% of males. More specifically, females reported higher difficulty in identifying and describing feelings and lower externally oriented thinking compared to males. Considering the low Cronbach’s alpha of the EOT subscale, this result should be not considered reliable. Nonetheless, it is possible to affirm that females reported higher difficulty in identifying and describing than males, while dissociation levels were similar in females and males, in line with the majority of studies conducted among adolescents [53]. Accordingly, about 27% of females and 26% of males crossed the cut-off point of the A-DES. Regarding age group differences, although some studies suggested that alexithymia [17,18] and dissociation levels [19,20] decreased during adolescence, we did not find any significant difference.

### 4.2. The Correlations between Internet Use, Alexithymia, and Dissociation

Now, moving on to discuss the role of alexithymia and dissociation on problematic internet use, and looking at the correlations, the results confirm that the higher the alexithymia and dissociation, the higher the Compulsive Internet Use will be. However, the strength of these associations is quite different between females and males. Among females, difficulty to identify feelings is the aspect of alexithymia most related to Compulsive Internet Use, while among males even the difficulty to describe and communicate one’s own feelings to others was important. These results confirmed that adolescents who have difficulties with identifying, expressing, and communicating emotions may overuse the internet to regulate their emotions better and to fulfill their unmet social needs. Another difference deserves our attention. All the aspects of dissociation, namely dissociative amnesia, absorption, and imaginative involvement, depersonalization and derealization, and passive influence were associated with Compulsive Internet Use, mostly among females. This relation in the whole sample can be explained in two ways. On the one hand, the tendency to use dissociative mechanisms (from normal to pathological levels) may find expression in the various activities on the internet, which can offer to users an experience of dissociative process. Therefore, internet use lends itself to use dissociative processes for manage emotions. On the other hand, internet usage can elicit dissociative experiences, such as absorption, immersion, and imaginative involvement, with experiences of unreality or detachment from one’s mind, self or body/one’s surroundings [62]. The stronger associations between dissociative aspects and Compulsive Internet Use among females compared to males, might be explained by the marked alexithymia present in this group. With regard to the association between alexithymia and dissociation, difficulty identifying feelings was the dimension mostly connected to dissociation, in both groups. Scholars argue that alexithymia (as a personality trait) fosters dissociation (as a defense mechanism/coping strategy/symptoms) [47]. However, conversely, because in dissociation one’s feelings are divided off from awareness, adolescents could have no words to describe what they are unaware of, or are too ashamed to talk about [46].

### 4.3. The Role of Dissociation on the Relation between Alexithymia and Problematic Internet Use

Given all of the above, the mediation moderated model with alexithymia as a predictor, dissociation as a mediator, Compulsive Internet Use as an outcome variable, and gender as a moderator, indicated that the effect of alexithymia on Compulsive Internet Use mediated by dissociation was moderated by gender, with dissociation mediating the effect only among females. Alexithymia predicted Compulsive Internet Use and dissociation both in females and males. Therefore, this confirms that difficulty in emotional regulation may induce dissociative mechanisms and problematic internet use. However, the effect of dissociation on Compulsive Internet Use, including alexithymia, was moderated by gender. The mediation effect of dissociation was significant only for females. Therefore, although dissociation levels were similar among males and females, only in the latter group did dissociation partially explain the effect of alexithymia on Compulsive Internet Use. This result might be explained by the differences between the two groups regarding alexithymia, Symptoms/Difficulties, and the use of internet.

### 4.4. Limitations

Before concluding, limitations must be highlighted. First, the present study considers a convenience sample of Italian adolescents. Therefore, this undermines the generalizability of the results. Second, all the measures were self-reported. Therefore, it is not possible to exclude the potential effects of social desirability, over/under reporting, etc., as well as common method variance. Third, although theoretically, clinically, and empirically it is well accepted that alexithymia has a role in both dissociation and Compulsive Internet Use, and dissociation has a role in Compulsive Internet Use, this study was cross-sectional, thus it was not possible to exclude possible bidirectional relations.

## 5. Conclusions

In conclusion, this study suggests that problematic internet use of females is increasing, or at least it is not less worrying compared to that of males. Adolescent females showed higher problematic internet use than males. They also reported higher alexithymia levels than males. Difficulty identifying and describing feelings and dissociation are risk factors for Compulsive Internet Use in adolescence, irrespective of gender. However, among females, dissociation played a key role in explaining Compulsive Internet Use levels. In fact, among females, the link between alexithymia and Compulsive Internet Use can be explained in part by the role that dissociation plays.

Future research should monitor and compare problematic internet use, alexithymia, and dissociation levels in male and female adolescents. The role of dissociation in the relationships between alexithymia and Compulsive Internet Use, taken separately for females and males, should be further investigated. The findings of this study should be replicated in other samples of adolescents, clinical and non-clinical, both in cross-sectional and longitudinal studies, as well as through cross-cultural studies.

This study suggests the need to carry out a prevention program on the risks related to Compulsive Internet Use. Moreover, prevention and treatment programs focusing on and aiming at reducing alexithymia (in particular the difficulty to identify feelings) and dissociation, and the promotion of emotion regulation strategies, should be developed to verify their efficacy in reducing the problematic internet use. On the basis of results coming from future research, policy and practice could be implemented to face the topical and relevant issue of problematic internet use in adolescents.

## Figures and Tables

**Table 1:** Descriptive statistics of demographic variables, Internet Use, Symptoms/Difficulties, and Satisfaction with Life.

	Early (*n* = 121)		Middle (*n* = 238)		Late (*n* = 235)				
	Female	Male	Female	Male	Female	Male	Test		
*N*	(*n* = 54)	(*n* = 67)	(*n* = 106)	(*n* = 132)	(*n* = 123)	*(n = 112)*	*n.s.*		
	*M ± SD*	*M ± SD*	*M ± SD*	*M ± SD*	*M ± SD*	*M ± SD*	G	G. A.	Int.
Listening to music	3.31 ± 0.82	2.84 ± 0.88	3.29 ± 0.75	3.01 ± 0.85	3.17 ± 0.82	3.37 ± 0.67	*	*n.s.*	*
	3.05 ± 0.88		3.14± 0.82		3.26 ± 0.75				
Watching videos/series	3.02 ± 0.81	3.00 ± 0.83	2.92 ± 0.81	2.94 ± 0.89	3.00 ± 0.85	3.25 ± 0.81	*n.s.*	*n.s.*	*n.s.*
	3.01 ± 0.82		2.93 ±0.85		3.12 ± 0.84				
Viewing images, photos, stories	2.96 ± 0.84	2.51 ± 0.99	3.12 ± 0.72	2.65 ± 0.83	3.16 ± 0.74	3.01 ± 0.75	*	*	*
	2.71 ± 0.95		2.89 ± 0.82		3.01 ± 0.75				
Gaming	1.89 ± 0.88	2.78 ± 0.98	1.91 ± 0.76	2.48 ± 0.88	1.80 ± 0.89	2.47 ± 1.00	**	*n.s.*	*n.s.*
	2.38 ± 1.03		2.22 ± 0.87		2.12 ± 1.00				
Searching for information	2.91 ± 0.68	2.64 ± 0.88	2.94 ± 0.55	2.97 ± 0.75	3.19 ± 0.70	3.00 ± 0.63	*	*	*n.s.*
	2.76 ± 0.81		2.96 ± 0.67		3.10 ± 0.67				
Studying	2.81 ± 0.73	2.67 ± 0.86	2.98 ± 0.70	2.73 ± 0.76	3.17 ± 0.74	2.72 ± 0.74	*	*n.s.*	*n.s.*
	2.74 ± 0.80		2.84 ± 0.74		2.96 ± 0.77				
Chatting with friends/acquaintances	3.43 ± 0.74	3.03 ± 0.87	3.57 ± 0.55	3.29 ± 0.78	3.58 ± 0.56	3.48 ± 0.57	*	*	*n.s.*
	3.21 ± 0.83		3.42 ± 0.70		3.54 ± 0.56				
Sending/receiving email	1.81 ± 0.80	1.55 ± 0.63	1.74 ± 0.65	1.69 ± 0.68	2.24 ± 0.76	1.89 ± 0.71	*	**	*n.s.*
	1.67 ± 0.72		1.71 ± 0.66		2.07 ± 0.76				
Navigating without a destination	2.06 ± 1.02	1.81 ± 0.89	2.37 ± 0.97	1.91 ± 0.95	2.41 ± 0.95	1.94 ± 0.88	*	*n.s.*	*n.s.*
	1.93 ± 0.95		2.11 ± 0.98		2.18 ± 0.94				
Shopping	1.91 ± 0.73	1.90 ± 0.78	2.24 ± 0.83	1.87 ± 0.82	2.41 ± 0.87	2.04 ± 0.78	*	*	*n.s.*
	1.90 ± 0.76		2.03 ± 0.85		2.23 ± 0.85				
Making new friends or dating	1.78 ± 0.86	1.70 ± 0.80	1.62 ± 0.83	1.60 ± 0.86	1.64 ± 0.83	1.75 ± 0.81	*n.s.*	*n.s.*	*n.s.*
	1.74 ± 0.82		1.61 ± 0.85		1.69 ± 0.82				
Time spent on the internet	2.78 ± 0.46	2.54 ± 0.78	2.80 ± 0.56	2.70 ± 0.52	2.75 ± 0.57	2.82 ± 0.41	*n.s.*	*n.s.*	*
	2.64 ± 0.67		2.74 ± 0.54		2.78 ± 0.50				
Symptoms/Difficulties	2.25 ± 0.69	1.53 ± 0.43	2.32 ± 0.68	1.75 ± 0.59	2.35 ± 0.61	1.91 ± 0.59	***	*	*n.s.*
	1.85 ± 0.66		2.01 ± 0.69		2.14 ± 0.64				
Satisfaction with Life	2.91 ± 0.63	2.98 ± 0.57	2.78 ± 0.57	2.89 ± 0.61	2.73 ± 0.52	2.79 ± 0.58	*n.s.*	*	*n.s.*
	2.95 ± 0.60		2.84 ± 0.59		2.76 ± 0.55				

Notes: G = Gender; G.A. = Group Age; Int. = Interaction between G and G.A. *n.s*. = Not significant; * significant at *p* < 0.05 with small effect size; ** significant at *p* < 0.05 with medium effect size; *** significant at *p* < 0.05 with large effect size.

**Table 2:** Gender differences in the mean scores of the TAS-20, A-DES, and CIUS-14 total scores.

	Females (*n* = 283)		Males (*n* = 311)		*F*	*p*	*ŋ_p_^2^*
	*M*	*SD*	*M*	*SD*			
TAS-20 TS	58.26	0.72	51.54	0.67	46.91	0.000	0.074
A-DES TS	2.90	0.12	2.66	0.11	2.17	0.141	0.004
CIUS-14	22.58	0.69	17.51	0.64	28.93	0.000	0.047

**Table 3:** Age group differences in the mean scores of the TAS-20, A-DES, and CIUS-14 total scores.

	Early (*n* = 121)		Middle (*n* = 238)		Late (*n* = 235)		*F*	*p*	*ŋ_p_^2^*
	*M*	*SD*	*M*	*SD*	*M*	*SD*			
TAS-20 TS	55.56	1.04	54.87	0.74	54.26	0.74	0.54	0.584	0.002
A-DES TS	2.97	0.17	2.69	0.13	2.69	0.13	0.97	0.381	0.003
CIUS-14	20.42	0.99	18.58	0.71	21.12	0.71	3.31	0.037	0.011

**Table 4:** Gender differences in the mean scores of the TAS-20 and A-DES subscales.

	Females (*n* = 283)		Males (*n* = 311)		*F*	*p*	*ŋ_p_^2^*
	*M*	*SD*	*M*	*SD*			
DIF	19.89	0.42	15.82	0.40	48.10	0.000	0.075
DDF	17.28	0.26	13.95	0.25	81.63	0.000	0.121
EOT	20.73	0.25	21.69	0.24	7.21	0.007	0.012
DA	2.73	0.12	2.55	0.12	1.01	0.317	0.002
AbII	2.86	0.12	2.82	0.12	0.07	0.797	0.001
DD	2.84	0.12	2.48	0.12	4.66	0.031	0.008
PI	3.12	0.13	2.86	0.13	2.03	0.155	0.003

Note: DIF = difficulty identifying feelings; DDF = difficulty describing feelings to others; EOT = externally oriented style of thinking; TAS-20 TS = TAS-20 total score; DA = dissociative amnesia; AbII = absorption and imaginative involvement; DD = depersonalization and derealization; PI = passive influence; A-DES TS = A-DES total score.

**Table 5:** Bivariate correlations of TAS-20, A-DES, and CIUS-14 as a function of gender.

	1	2	3	4	5	6	7	8	9	10
1. DIF	-	0.67 *	−0.01	0.87 *	0.34 *	0.34 *	0.48 *	0.40 *	0.45 *	0.38 *
2. DDF	0.70 *	-	0.02	0.81 *	0.16 *	0.08	0.23 *	0.15 *	0.19 *	0.24 *
3. EOT	−0.10	−0.13 *	-	0.39 *	0.19 *	0.21 *	0.11	0.13 *	0.16 *	0.13 *
4. TAS TS	0.90 *	0.80 *	0.25 *	-	0.34 *	0.32 *	0.43 *	0.35 *	0.41 *	0.38 *
5. DA	0.38 *	0.22 *	0.23 *	0.42 *	-	0.78 *	0.78 *	0.77 *	0.91 *	0.30 *
6. AbII	0.35 *	0.20 *	0.19 *	0.38 *	0.86 *	-	0.76 *	0.74 *	0.88 *	0.32 *
7. DD	0.44 *	0.26 *	0.16 *	0.45 *	0.88 *	0.83 *	-	0.82 *	0.95 *	0.33 *
8. PI	0.44 *	0.25 *	0.12 *	0.43 *	0.83 *	0.78 *	0.84 *	-	0.90 *	0.33 *
9. A-DES TS	0.43 *	0.25 *	0.19 *	0.45 *	0.95 *	0.92 *	0.97 *	0.91 *	-	0.35 *
10. CIUS-14	0.44 *	0.35 *	0.02	0.44 *	0.23 *	0.26 *	0.26 *	0.22 *	0.26 *	-

Note: Correlations for female participants (*n* = 283) are presented above the diagonal, and correlations for male participants (*n* = 311) are presented below the diagonal. DIF = difficulty identifying feelings; DDF = difficulty describing feelings to others; EOT = externally oriented style of thinking; TAS-20 TS = TAS-20 total score; DA = dissociative amnesia; AbII = absorption and imaginative involvement; DD = depersonalization and derealization; PI = passive influence; A-DES TS = A-DES total score. * *p* < 0.05.

**Table 6:** Standardized coefficients of the moderated mediational model.

Paths	Direct Effects				Indirect Effects	
	*b*	*se*	*t*	*p*	*b*	*CI (95%)*
Total sample (N = 594) TAS-20 TS →A-DES TS Gender →A-DES TS TAS-20 TS x Gender →A-DES TS A-DES TS →CIUS-14 Gender →CIUS-14 A-DES TS x Gender →CIUS-14 TAS-20 TS →CIUS-14 TAS-20 TS x Gender →CIUS-14	2.232 7.099 0.597 0.029 −2.921 −0.035 0.332 0.132	0.190 4.472 0.380 0.007 0.845 0.015 0.040 0.079	11.69 1.59 1.57 3.83 −3.46 −2.28 8.30 1.66	<0.001 0.112 0.117 <0.001 <0.001 0.023 <0.001 0.096		
Females (*n* = 283) A-DES TS →CIUS-14 TAS-20 TS →CIUS-14	0.048 0.262	0.011 0.053	4.23 4.89	<0.001 <0.001	0.093	0.093, 0.158
Males (*n* = 311) A-DES TS →CIUS-14 TAS-20 TS →CIUS-14	0.012 0.395	0.010 0.058	1.21 6.72	0.224 <0.001	0.032	−0.024, 0.090

Note: → = effect on; x = interaction with.

## Data Availability

Data are available from the corresponding author on reasonable request.
